# Biotic and abiotic factors influencing the prevalence, intensity and distribution of *Eucoleus aerophilus* and *Crenosoma vulpis* in red foxes, *Vulpes vulpes* from Romania

**DOI:** 10.1016/j.ijppaw.2020.05.009

**Published:** 2020-05-28

**Authors:** Georgiana Deak, Călin Mircea Gherman, Angela Monica Ionică, Áron Péter, D. Attila Sándor, Andrei Daniel Mihalca

**Affiliations:** a Department of Parasitology and Parasitic Diseases, University of Agricultural Sciences and Veterinary Medicine of Cluj-Napoca, Calea Mănăștur 3-5, 400372, Cluj-Napoca, Romania; b Molecular Biology and Veterinary Parasitology Unit (CDS 9), “Regele Mihai I al României” Life Science Institute, University of Agricultural Sciences and Veterinary Medicine of Cluj-Napoca, 400372, Calea Mănăștur 3-5, Cluj-Napoca, Romania; c Department of Parasitology and Zoology, University of Veterinary Medicine, István u. 2, 1078, Budapest, Hungary

**Keywords:** *Crenosoma vulpis*, *Vulpes vulpes*, *Eucoleus aerophilus*, Romania, Nematodes

## Abstract

To understand the importance of host’ habitat choice in determining parasite burden, we studied the distribution of two helminth parasites of the red fox (*Vulpes vulpes*) in south-eastern Europe (Romania): *Crenosoma vulpis* and *Eucoleus aerophilus*, both widely distributed respiratory nematodes parasitic in various carnivores. Even though the life cycle and biology of the two nematodes follow a different pattern, both parasites appear to be co-distributed and often co-infect foxes with variable prevalences across their range. Between July 2016 and August 2018, 550 red foxes, *V. vulpes* were collected by hunters in different localities from 22 counties of Romania and examined by necropsy. All parasites found in the trachea and bronchial system were collected and preserved in 70% ethanol. We characterised red fox/parasite habitats using seven predictors (fragmentation, altitude, presence/absence of water surface, per cent cover of arable land/grassland/urbanized areas/forest cover/wetlands). Prevalence, abundance, intensity, and sex ratio were calculated and statistically analysed using the R software. Out of the 550 examined foxes, 76.2% were infected with lungworms. The overall prevalence was 32.0% for *C. vulpis* and 72.5% for *E. aerophilus*. The mean intensity of infection was 13.70 for *C. vulpis* 6.15 for *E. aerophilus*. For both nematodes, the prevalence was significantly higher in males than in females, and there was no influence of hosts’ age. No statistical differences were found for intensity and mean intensity in the case of infection with *C. vulpis* and *E. aerophilus* between age and sex categories. The abundance of *C. vulpis* showed a strong positive relationship with the presence of wetlands and habitat fragmentation. We found a significant correlation between the abundance of *E. aerophilus* and altitude, with foxes from higher elevations showing higher prevalences.

## Highlights

• *Crenosoma vulpis* and *Eucoleus aerophilus* are widely distributed respiratory nematodes.
• Prevalence, abundance, intensity were calculated and statistically analysed using R software Grs.
• There was a significant correlation between the abundance of *E. aerophilus* and altitude.
• The abundance of *C. vulpis* showed a strong positive relationship with the presence of wetland.

## Graphical abstract

**Figure:**
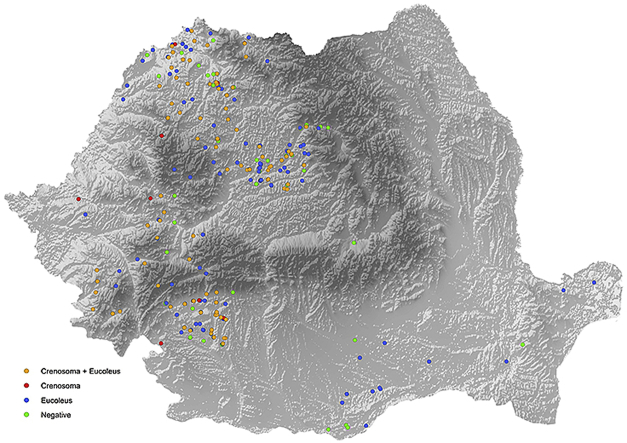

## 1 Background

Parasitic helminths are common in most carnivore species and may cause significant pathology in their hosts. As most species are transmitted via food ingestion (either from an intermediate host or accidentally), host diet and habitat use may play key roles in the acquisition of infections (Samuel et al., 2001). For generalist carnivores, the diet is determined primarily by the habitat choice and may show wide seasonal fluctuations in temperate regions (Rosalino et al., 2011). To understand the importance of host’ habitat choice in determining parasite burden, we studied the distribution of *Crenosoma vulpis* and *Eucoleus aerophilus* (=*Capillaria aerophila*), two widely distributed respiratory nematodes of the red fox (*Vulpes vulpes*), in south-eastern Europe, Romania.

*Crenosoma vulpis* is an ovoviviparous strongylid, infecting the bronchioles, bronchi and sometimes trachea of a wide range of canids (*Canis lupus*, *Canis aureus*, *Nyctereutes procyonoides, Urocyon cinereoargenteus, Vulpes lagopus*, *V. vulpes*) and mustelids (*Lutra lutra*, *Martes* spp., *Meles meles*) in Europe and North America (Anderson, 2000; Gherman and Mihalca, 2017). Its life cycle is heteroxenous and includes multiple species of terrestrial gastropods as intermediate hosts (Anderson, 2000). Contamination of the carnivore definitive hosts is by ingesting snails containing infective L3 larvae (Anderson, 2000). Unlike the case of other lungworms of carnivores, for which various small vertebrates have been demonstrated as paratenic hosts (Mozzer and Lima, 2015; Colella et al., 2019), no such information is available for *C. vulpis* (Colella et al., 2016).

*Eucoleus aerophilus* is an oviparous capillariid, infecting mainly the tracheal and bronchial mucosa of canids, felids and mustelids worldwide (Anderson, 2000). The eggs embryonate and develop in the environment, to become infective and probably need to be eaten by an earthworm to contaminate a carnivore finally (Anderson, 2000). Even though *E. aerophilus* is a relatively common parasite of carnivores, its life cycle is still poorly understood, and the obligate or facultative role of earthworms is still uncertain (Traversa et al., 2011).

Even though the life cycle and biology of the two nematodes follow a different pattern, both parasites appear to be co-distributed and often co-infect foxes. The prevalences of *C. vulpis* and *E. aerophilus* show wide variability and distribution seems to be patchy across their range (Nevárez et al., 2005; Davidson et al., 2006; Hodžić et al., 2016; Schug et al., 2018). Only a few studies have evaluated the influence of environmental factors on the spatial distribution of these respiratory nematodes (Tolnai et al., 2015; Maksimov et al., 2017; Čabanová et al., 2018). However, our understanding on the drivers of distribution and environmental risk factors associated with lungworm infection in carnivores is still limited, despite their very common occurrence.

Our study aimed to analyse the potential environmental and host-related (biotic) factors which can influence the epidemiological features (presence, prevalence, intensity) of *C. vulpis* and *E. aerophilus* infection in red foxes.

## 2 Materials and methods

### 2.1 Parasite data

**Fig. 1:**
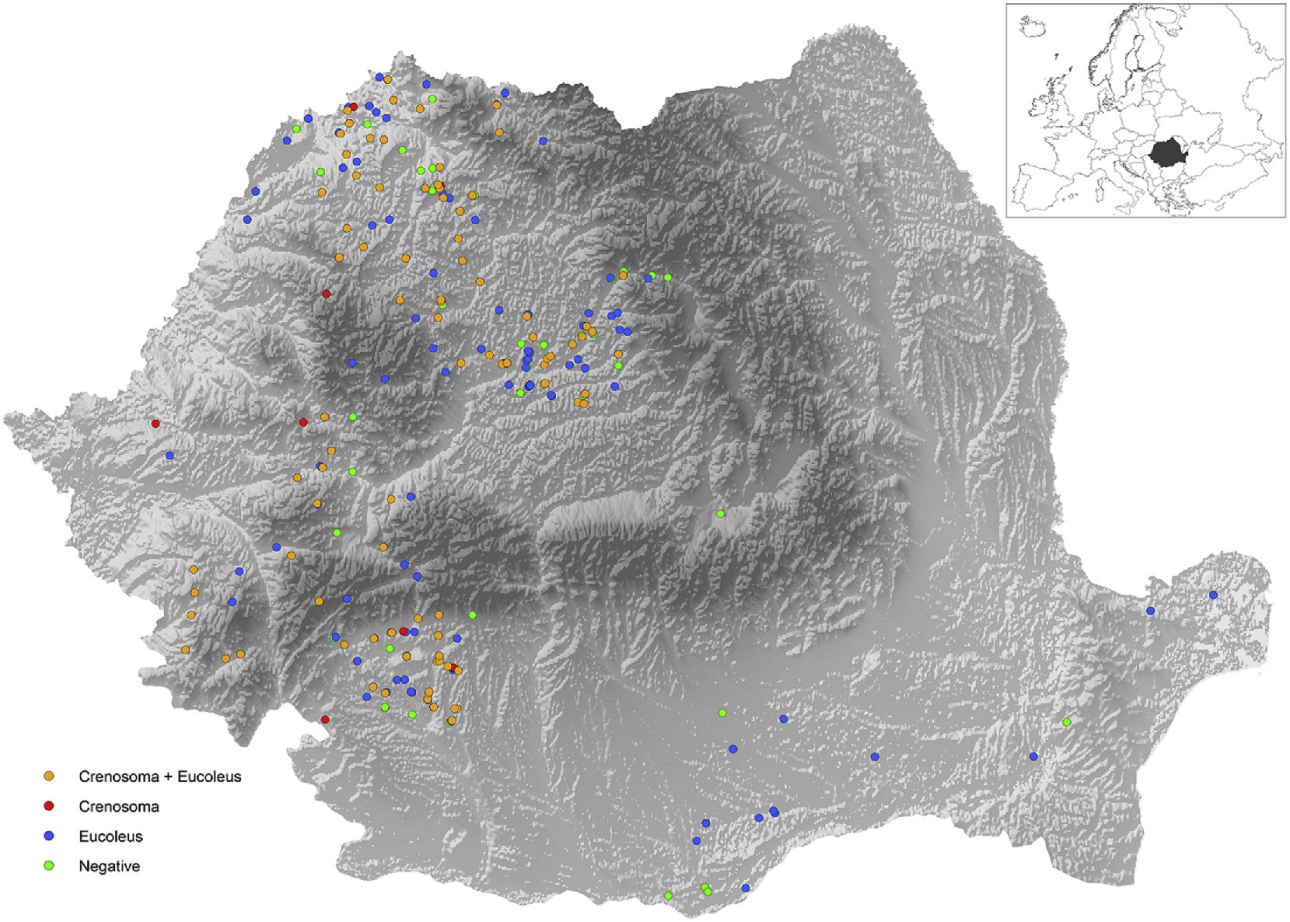
Distribution of *Crenosoma vulpis* and *Eucoleus aerophilus* in Romania.

Between July 2016 and August 2018, 550 red foxes (315 males, 235 females; 180 young, 360 adults) were collected by hunters in different localities from 22 counties of Romania, through the country veterinary authority, as part of the rabies surveillance program (supplementary material) (Fig. 1). Romania is a climatically and geographically heterogeneous country, with the topography almost evenly divided among mountains (31%), plains (33&), and hills (36%) and five types of climate (alpine, cool continental, wet temperate continental, wet warm continental and warm oceanic) (http://www.meteoromania.ro/clima/clima-romaniei/). All foxes originated from areas with wet continental climates.

For safety reasons, only foxes negative for rabies have been used for the present work. For each fox, the location (county, locality, precise location if available), age (young, less than one-year-old; adult, more than one-year-old, according to Harris, 1978) and sex were recorded. During the necropsy, the respiratory tract was longitudinally opened and carefully examined for nematodes under a stereo zoom microscope. All parasites found in the trachea and bronchial system were collected and preserved in 70% ethanol. All parasites were identified to species level and sexed using keys and morphological descriptions (Anderson et al., 2009; Latrofa et al., 2015).

### 2.2 Environmental predictors

The collection locations were geo-referenced and environmental predictors were collected for each point, using a 2 × 2 km cell grind containing the geo-referenced coordinates of the collection site (Sándor et al., 2017). These cells (area = 400 ha) are similar to the average red-fox home range size in semi-natural and natural habitats in Europe (mean 413.42; min = 12.95, max = 1990.00; SD 393.1192 ha, n = 84 studies, (Lucherini and Lovari, 1996; Bartoń and Zalewski, 2007; Holmala and Kauhala, 2008; Šálek et al., 2015).

We characterised red fox/parasite habitats using seven predictors (fragmentation, altitude, presence/absence of water surface, per cent cover of arable land/grassland/urbanized areas/forest cover). Fragmentation was an integer number, assigned for each cell using the number of land-cover units crossed by the NW-SE diagonal at the cell level. The CORINE LandCover (European Environment Agency, http://www.eea.europa.eu/) dataset was used as source for land-use data (see Table 1 for the associated CORINE LandCover categories). There was no statistical difference (*x*^2^ = 0.2257956, df = 3 p < 0.97) between land-use composition inside the sampled 2 × 2 km plots and the overall land-use composition of the region, thus we consider that our results may be generalized for the whole region. Altitude for each cell was extracted from WorldClim database (www.worldclim.org), the data being downloaded from WorldClim database with the highest resolution provided, of 30 arc-seconds (one pixel equals ca. 0.6 km^2^).

**Table 1:** Correspondence between CORINE LandCover categories and land-use types used in this study.

Code Level 3	Label Level3	Assigned land-use type
**111**	Continuous urban fabric	Urban
**112**	Discontinuous urban fabric	Urban
**121**	Industrial or commercial units	Urban
**122**	Road and rail networks and associated land	Urban
**123**	Port areas	Urban
**124**	Airports	Urban
**131**	Mineral extraction sites	Urban
**132**	Dump sites	Urban
**133**	Construction sites	Urban
**141**	Green urban areas	Urban
**142**	Sport and leisure facilities	Urban
**211**	Non-irrigated arable land	Arable
**212**	Permanently irrigated land	Arable
**213**	Rice fields	Arable
**221**	Vineyards	Arable
**222**	Fruit trees and berry plantations	Arable
**223**	Olive groves	Arable
**231**	Pastures	Grassland
**241**	Annual crops associated with permanent crops	Arable
**242**	Complex cultivation patterns	Arable
**243**	Land principally occupied by agriculture, with significant areas of natural vegetation	Grassland
**244**	Agro-forestry areas	Forest
**311**	Broad-leaved forest	Forest
**312**	Coniferous forest	Forest
**313**	Mixed forest	Forest
**321**	Natural grasslands	Grassland
**322**	Moors and heathland	Grassland
**323**	Sclerophyllous vegetation	Grassland
**324**	Transitional woodland-shrub	Forest
**333**	Sparsely vegetated areas	Grassland

### 2.3 Statistical procedures

Prevalence, abundance, intensity, and sex ratio were calculated and statistically analysed using the R software (Version 3.2.3). Sample prevalence data were analysed using Fisher's exact test. Relationship between parasite prevalence and environmental predictors (land-use and altitude) was tested using Spearman Rank Correlation. Differences were considered significant when p < 0.05.

## 3 Results

Three species of pulmonary nematodes have been identified: *Angiostrongylus vasorum*, *Crenosoma vulpis* and *Eucoleus aerophilus*. The data regarding the prevalence and intensity of *A. vasorum* were presented elsewhere (Deak et al., 2017). Out of the 550 examined foxes, 419 (76.2%) were infected with *C. vulpis* or *E. aerophilus* (Table 2). The overall prevalence was 32.0% for *C. vulpis* and 72.5% for *E. aerophilus*. The intensity of infection varied between 1 and 265 nematodes per fox for *C. vulpis* (mean intensity 13.70) (Table 3) and between 1 and 51 nematodes per fox for *E. aerophilus* (mean intensity 6.15) (Table 4). For both *C. vulpis* and *E. aerophilus*, the prevalence was significantly higher in males than in females, and there was no influence of the age (Table 5, Table 6). Male foxes also had significantly more co-infections than females (Table 7). No statistical differences were found for intensity and mean intensity in the case of infection with *C. vulpis* and *E. aerophilus* between age and sex categories (Table 2, Table 3).

**Table 2:** Prevalence of *Crenosoma vulpis* and *Eucoleus aerophilus* in red foxes from Romania.

Category	Examined	Negative	*C. vulpis*	*E. aerophilus*	*C. vulpis* only	*E. aerophilus* only	*C. vulpis* + *E. aerophilus*
**Total**	550	131	176	399	20	243	156
		(23.8%)	(32.0%)	(72.5%)	(3.6%)	(44.2%)	(28.4%)
**Males**	315	60	115	244	11	140	104
		(19.0%)	(36.5%)	(77.5%)	(3.5%)	(44.4%)	(33.0%)
**Females**	235	71	61	155	9	9	52
		(30.2%)	(26.0%)	(66.0%)	(3.8%)	(3.8%)	(22.1%)
**Young**	180	37	68	134	9	75	59
		(20.6%)	(37.8%)	(74.4%)	(5.0%)	(42.7%)	(32.8%)
**Adult**	370	94	108	265	11	168	97
		(25.4%)	(29.2%)	(71.6%)	(3.0%)	(45.4%)	(26.2%)

**Table 3:** Intensity of *Crenosoma vulpis* in red foxes from Romania and its statistical interpretation (calculated for 168 foxes out the 176 infected ones).

Fox category		Male nematodes	Female nematodes	Total	M:F ratio	Min	Median	Max	Mean intensity ± STDEV	H
**Sex**	Male	411	1303	1714	1:3.17	1	2	265	15.58 ± 32.09	3.27; n.s.
	Female	165	424	589	1:2.57	1	2	87	10.16 ± 17.28	
**Age**	Young	337	919	1256	1:2.73	1	5	265	19.03 ± 38.80	2.35, n.s.
	Adult	239	808	1047	1:3.38	1	3	115	10.26 ± 17.07	
**Total**		**576**	**1727**	**131**	**1:2.99**	**1**	**4**	**265**	**13.70 ± 27.94**	

Significance levels: n.s. = not significant.

**Table 4:** Intensity of *Eucoleus aerophilus* in red foxes from Romania and its statistical interpretation (calculated for 378 foxes out the 399 infected ones).

Fox category		Male nematodes	Female nematodes	Total	M:F ratio	Min	Median	Max	Mean intensity ± STDEV	H
**Sex**	Male	338	1255	1593	1:3.71	1	3.5	24	4.94 ± 4.37	3.82; *
	Female	154	578	732	1:3.75	1	4	51	6.93 ± 7.51	
**Age**	Young	186	632	818	1:3.40	1	4	28	6.29 ± 5.98	0.36; n.s.
	Adult	306	1201	1507	1:3.92	1	4	51	6.08 ± 6.81	
**Total**		**492**	**1833**	**2325**	**1:3.73**	**1**	**4**	**51**	**6.15 ± 6.53**	

Significance levels: p < ‘*’ 0.05, n.s. = not significant.

**Table 5:** Prevalence of *Crenosoma vulpis* by sex and age of foxes (n = 550 foxes; 176 infected);32%; 95% CI = 28.24–36.01%) and its statistical interpretation.

Fox category		%	95% CI	*Χ*^2^
**Sex**	Male	36.51	31.38–41.96	6.40; **
	Female	25.96	20.48–32.06	
**Age**	Young	37.78	30.67–45.29	3.72; 0.053
	Adult	29.19	24.79–34.02	

Significance levels: p < ‘**’ 0.01.

**Table 6:** Prevalence of *Eucoleus aerophilus* by sex and age of foxes (n = 550 foxes; 399 infected; 72.55%; 95% CI = 68.67–76.11%) and its statistical interpretation.

Fox category		%	95% CI	*Χ*^2^
**Sex**	Male	77.46	72.53–81.73	8.373; **
	Female	65.96	59.51–71.99	
**Age**	Young	74.44	67.42–80.64	0.353; n.s.
	Adult	71.62	66.82–75.97	

Significance levels: p < ‘**’ 0.01, n.s. = not significant.

**Table 7:** Statistical analysis of co-infection rates of foxes with *C. vulpis* and *E. aerophilus*.

Fox category		%	95% CI	*Χ*^2^
**Sex**	Male	33.02	28.05–38.39	7.33; **
	Female	22.13	16.99–27.98	
**Age**	Young	32.78	25.98–40.15	2.25; n.s.
	Adult	26.22	22.00–30.93	

Significance levels: p < ‘**’ 0.01, n.s. = not significant.

Some of the foxes were received without exact collecting locations (n = 196, 35.64%), and these were excluded from the habitat-related analyses. The abundance of *C. vulpis* showed a strong positive relationship with the presence of wetlands and was so significant that all the other habitat predictors showed a negative correlation (Table 8). Both, prevalence (*z* = 4.440, p < 0.01) and intensity (*z* = 4.668, p < 0.01) were linked to the presence of wetlands. Habitat fragmentation (number of different land-use patches inside the 400 ha plot) showed a positive correlation with *C. vulpis* abundance. However, fragmentation was uncorrelated to the number or surface area of wetlands (*z* = 0.0023, p > 0.1).

**Table 8:** Effect of environmental predictors (relative area of land-use categories, altitude and fragmentation inside 400 ha sample plots) on the abundance of the nematode *Crenosoma vulpis* in red foxes from Romania.

		Estimate	Std. Error	z value	Sign
(Intercept)		280.0388	59.9906	4.668	
Arable		−367.1515	80.5074	−4.560	***
Forest		−512.0818	116.1698	−4.408	***
Grassland		−285.7888	61.5950	−4.640	***
Urban		0.1270	2.3779	0.053	
Wetlands		167.8188	85.8575	4.440	***
Altitude		0.2429	0.2318	1.048	
Fragmentation	84.6652	15.8377	5.346	***	

Significance levels: p < ‘***’ 0.001, ‘**’ 0.01, ‘*’ 0.05.

We found a significant correlation between the abundance of *E. aerophilus* and altitude (z = 1.977, p = 0.0480), with foxes from higher elevations showing higher prevalences (independent of host sex or age). There was no relationship, however between altitude and the intensity of parasitism.

## 4 Discussion

The data on the influence of host-related factors (sex, age) on the prevalence, intensity and abundance of *C. vulpis* and *E. aerophilus* show wide variations. Several studies have shown that males foxes are more commonly infected with *C. vulpis* than females (Goble and Cook, 1942; Willingham et al., 1996), while others did not find any differences between sexes (Jeffery et al., 2004). Similarly, *E. aerophilus* was found more commonly in males foxes than in females (Morgan et al., 2008). Our study confirms this characteristic also in Romania, for both parasites. The higher infection rate of males has been attributed to several reasons such as increased susceptibility to infection due to higher testosterone levels, increased exposure to parasites due to more risky behaviour, different diet due to their increased body size (Schmid-Hempel, 2011) or longer distances travelled predominantly by sub-adult males (Walton et al., 2018). It is uncertain why *E. aerophilus* had higher mean intensity in female foxes compared to males. It is speculated that variation in habitat use, foraging time, and/or diet may influence the acquisition of helminth species (Bush, 1990; Fedynich et al., 2005). Consecutively, different exposure probabilities to infective parasitic stages will result in different host-sex related intensities.

Only a few studies have investigated the possible influence of environmental factors on the distribution and infection rate of *C. vulpis* and *E. aerophilus*. Moreover, results are not consistent between these studies, although all were geographically located in Central Europe (Tolnai et al., 2015; Maksimov et al., 2017; Čabanová et al., 2018). The only environmental factor which seems to influence the abundance of *C. vulpis* in our study was the presence of wetlands. Tolnai et al. (2015) showed that the infection rate with *C. vulpis* in foxes from Hungary was positively correlated only with annual precipitation. Maksimov et al. (2017) investigated the risk factors associated with shedding of *C. vulpis* larvae in the faeces of dogs in Germany and concluded that larval shedding is higher in the winter and higher in dogs less than one-year-old. The higher proportion of moorlands was the most important among the environmental factors associated with a higher risk for *C. vulpis* larval shedding in domestic dogs in Germany. Agricultural fields and water bodies were associated with a lower risk (Maksimov et al., 2017), which is in contrast to our results. Čabanová et al. (2018) concluded that the distribution of *C. vulpis* in foxes from Slovakia is not significantly influenced by any of the 16 environmental factors used as predictor variables of the regression modelling. Environmental factors do not necessarily affect the distribution and infection rate of *C. vulpis*, which is a heteroxenous nematode. They could act on the snail populations, intermediate hosts, thus indirectly impacting the nematode’ distribution and infection rate. Generally, relative air and soil humidity, atmospheric and soil temperature, as well as litter depth, influence the distribution of land snails (Nunes and Santos, 2012). Wetlands and moorlands, characterized by a high level of humidity, meet optimal conditions for increased snail populations' sizes.

Except for the altitude, no influence of other environmental factors was found in the case of prevalence, intensity and abundance of *E. aerophilus* in our study. The current knowledge on the biology and transmission dynamics of *E. aerophilus* to foxes does not allow to produce any strong hypothesis on why foxes at higher altitudes had higher prevalences. Several factors such as parasite infectivity and survival in the environment or different dietary preferences and behavior of foxes could be a possible cause. However, the infection rate with *E. aerophilus* was positively correlated with annual precipitation and negatively correlated with the mean annual temperature in a survey from Hungary (Tolnai et al., 2015). As shown in Fig. 1, *C. vulpis* was mostly absent from the lowlands (south east and the western border with Hungary). In spite that our results suggest that the altitude, annual precipitation, and temperature seems to influence prevalence, intensity and abundance of *E. aerophilus*, others appreciated that drivers influencing the occurrence of this parasite are still unknown (Traversa and Di Cesare, 2014). Moreover, the eggs of *E. aerophilus* develop in the environment and the role of earthworms in the life cycle is questionable. More recent data indicate that the life cycle of *E. aerophilus* is direct, with a facultative, not mandatory, intervention of earthworms (Bowman, 2002; Taylor et al., 2007).

Our study is the first to analyse and demonstrate the influence of the habitat fragmentation, which was positively correlated with *C. vulpis* abundance. In conclusion, the present study shows the common occurrence of *C. vulpis* and *E. aerophilus* in foxes from Romania and demonstrates the influence of only a few environmental factors on their abundance and no influence of most other environmental factors. This might be related to the ubiquity of foxes, their variable diet and the complex biology of these nematodes.

## Founding sources

This research did not receive any specific grant from funding agencies in the public, commercial, or not-for-profit sectors.
